# First genomic study on Lake Tanganyika sprat *Stolothrissa tanganicae*: a lack of population structure calls for integrated management of this important fisheries target species

**DOI:** 10.1186/s12862-018-1325-8

**Published:** 2019-01-08

**Authors:** Els L. R. De Keyzer, Zoë De Corte, Maarten Van Steenberge, Joost A. M. Raeymaekers, Federico C. F. Calboli, Nikol Kmentová, Théophile N’Sibula Mulimbwa, Massimiliano Virgilio, Carl Vangestel, Pascal Masilya Mulungula, Filip A. M. Volckaert, Maarten P. M. Vanhove

**Affiliations:** 1 Laboratory of Biodiversity and Evolutionary Genomics, KU Leuven, Charles Deberiotstraat 32, B-3000 Leuven, Belgium; 2 Capacities for Biodiversity and Sustainable Development (CEBioS), Operational Directorate Natural Environment, Royal Belgian Institute of Natural Sciences, Vautierstraat 29, B-1000, Brussels, Belgium; 3 Joint Experimental Molecular Unit & Biology Department, Royal Museum for Central Africa, Leuvensesteenweg 13, B-3080 Tervuren, Belgium; 4 Joint Experimental Molecular Unit & Operational Directorate Taxonomy and Phylogeny, Royal Belgian Institute of Natural Sciences, Vautierstraat 29, B-1000 Brussels, Belgium; 5 Faculty of Bioscience and Aquaculture, Nord University, Universitetsalléen 11, N-8026 Bodø, Norway; 6 Department of Botany and Zoology, Faculty of Science, Masaryk University, Kotlářská 2, CZ-611 37 Brno, Czech Republic; 7 Département de Biologie, Centre de Recherche en Hydrobiologie, B.P. 73, Uvira, Democratic Republic of Congo; 8 Zoology Unit, Finnish Museum of Natural History, University of Helsinki, P.O.Box 17, FI-00014 Helsinki, Finland; 9 Hasselt University, Centre for Environmental Sciences, Research Group Zoology: Biodiversity & Toxicology, Agoralaan Gebouw D, B-3590 Diepenbeek, Belgium

**Keywords:** Fish, Freshwater, High-throughput sequencing, RAD sequencing, SNP, Panmixis, Population genomics, East Africa, Great Lakes, Stock management

## Abstract

**Background:**

Clupeid fisheries in Lake Tanganyika (East Africa) provide food for millions of people in one of the world’s poorest regions. Due to climate change and overfishing, the clupeid stocks of Lake Tanganyika are declining. We investigate the population structure of the Lake Tanganyika sprat *Stolothrissa tanganicae,* using for the first time a genomic approach on this species. This is an important step towards knowing if the species should be managed separately or as a single stock. Population structure is important for fisheries management, yet understudied for many African freshwater species. We hypothesize that distinct stocks of *S. tanganicae* could be present due to the large size of the lake (isolation by distance), limnological variation (adaptive evolution), or past separation of the lake (historical subdivision). On the other hand, high mobility of the species and lack of obvious migration barriers might have resulted in a homogenous population.

**Results:**

We performed a population genetic study on wild-caught *S. tanganicae* through a combination of mitochondrial genotyping (96 individuals) and RAD sequencing (83 individuals)*.* Samples were collected at five locations along a north-south axis of Lake Tanganyika. The mtDNA data had low global FST and, visualised in a haplotype network, did not show phylogeographic structure. RAD sequencing yielded a panel of 3504 SNPs, with low genetic differentiation (F_ST_ = 0.0054; 95% CI: 0.0046–0.0066). PCoA, fineRADstructure and global F_ST_ suggest a near-panmictic population. Two distinct groups are apparent in these analyses (F_ST_ = 0.1338 95% CI: 0.1239,0.1445), which do not correspond to sampling locations. Autocorrelation analysis showed a slight increase in genetic difference with increasing distance. No outlier loci were detected in the RADseq data.

**Conclusion:**

Our results show at most very weak geographical structuring of the stock and do not provide evidence for genetic adaptation to historical or environmental differences over a north-south axis. Based on these results, we advise to manage the stock as one population, integrating one management strategy over the four riparian countries. These results are a first comprehensive study on the population structure of these important fisheries target species, and can guide fisheries management.

**Electronic supplementary material:**

The online version of this article (10.1186/s12862-018-1325-8) contains supplementary material, which is available to authorized users.

## Introduction

Freshwater ecosystems support more species per unit area than any other ecosystem. Yet, they currently suffer from fast declines in species richness [1]. The decline in biodiversity reduces the resilience of aquatic ecosystems, decreasing their ability to provide ecosystem services such as food, drinking water, climate regulation, and social and health benefits [2]. As freshwater habitats play an important role in fisheries with almost 13% of the world’s aquatic catches [3], and one third of African fish catches [4], this decrease in resilience jeopardizes the future of human communities [5]. Therefore, it is unfortunate that freshwater fisheries have been less well studied compared to marine fisheries and are often overlooked in policy and regulation matters [6].

The sustainable exploitation of freshwater ecosystem services benefits from science-based management, based on sound biological knowledge of the system and its species. An important component of biological information is related to the structure of fish populations. The genetic structure of fish populations can be used to support the delineation of demographic units [7, 8], commonly referred to as stocks. Knowledge about stocks allows to preserve genetic variation and to decide on the size of meaningful management units [9]. Currently, most fisheries management units are not sufficiently supported by information on the population structure of the target species [10, 11]. Lack of scientifically supported management entails a risk for overfishing, and loss of population densities [12], especially when catch effort is not spread homogeneously [11].

In tropical systems, the biological knowledge on fisheries target species is less advanced and information on the population structure is often lacking. Hence, the scope for science-based management is small. This also holds for the Great Lakes of East Africa, in spite of their ecological, economic and social significance. Lake Tanganyika (LT) is the oldest African Great Lake, in which unique and very diverse aquatic communities have evolved [13]. It is situated in the western range of the Great African rift valley, measures almost 680 km in length and 50 km in width, and contains more than 1.89 × 10^7^ km^3^ of water [14]. The oxygenated layer is deeper in the South (180 m) than in the North (120 m), as recorded during a dry season sampling [15]. The prevailing south-eastern winds cause an inclination of the thermocline, causing the upper water column to be somewhat warmer in the North (average annual temperature 25.8 °C), than in the South (average annual temperature 24 °C) [15, 16]. These differences are more pronounced in the dry season from May until September [16]. The lake is divided into three subbasins, which have been intermittently disconnected during periods of low water levels during its 6 million year history, forming distinct palaeolakes. The presumed prolonged division of the lake into these palaeolakes, approximately 1 million years ago, had profound influences on the lake’s diverse benthic fauna [17]. Lake levels continued to rise and fall, but it is assumed that since 106.000 years ago (106 kya), the subbasins of Lake Tanganyika have remained connected [18].

The fishery of LT plays an invaluable role in food security in one of the poorest regions in the world. Many people living near the lakeshore depend on artisanal fishing for their protein supply [19]. The Lake’s pelagic fisheries have a huge importance to local communities by providing almost 200,000 tons of fish yearly [20]. Pelagic catches are composed of mainly three species. The clupeids *Stolothrissa tanganicae* (Lake Tanganyika sprat; Clupeidae; Actinopterygii) and *Limnothrissa miodon* (Lake Tanganyika sardine; Clupeidae; Actinopterygii) provide 65% of the catch (by weight) [20]; a perciform predator, *Lates stappersii* (sleek lates; Latidae; Actinopterygii)*,* provides 30% of the catch [20]. Additionally, *S. tanganicae* serves as an important food source for *L. miodon* and *L. stappersii* [21]. In the northern part of LT, *S. tanganicae* dominates the catches of artisanal fishermen [22]. In the South, the species is less abundant and catches are dominated by *L. stappersii* [23]. *Stolothrissa tanganicae* has a life style that is reminiscent of that of marine clupeids. It forms schools that differ in size and density throughout the day [24]. The species migrate deeper into the lake at dawn and back to the surface at dusk, probably following their zooplankton prey [21] and escaping their predators. The fish live up to 1.5 years, reach maturity at about 70 mm standard length (SL) [25] and their maximum SL is about 100 mm [23]. *Stolothrissa tanganicae* spawns throughout the year, with peaks in February–May [26] or August–September [27] in the North of the lake and in August–December [28], and possibly April–July [29] in the South. Eggs are spawned pelagically, sink and hatch one to 1.5 days later before they have reached the anoxic zone [24]. Feeding habits have mostly been studied in the northern part of the lake, where *S. tanganicae* feeds on zooplankton, mainly the calanoid copepod *Tropodiaptomus simplex* [30].

Observations at landing sites have shown a decrease of clupeid catches in LT [31, 32]. Hence, multiple calls for better management of this unique resource have been made [33, 34]. Yet, prior to this, it is necessary to understand the genetic structure of the two species, as it is unclear if they should be treated as single stocks or to be managed as different populations. A collapse of the clupeid fisheries would threaten the food security of millions of people. Additionally, loss of clupeid fisheries will also harm the biodiversity in LT as people will turn to fishing less resilient species, such as littoral cichlids. Furthermore, agriculture could increase further to compensate for the loss of protein source, which will cause runoff, destroying important habitats. Overall, the fisheries of LT are data-poor, which hampers the assessment of the exploitation status of the targeted stocks [35]. Clupeids can be considered very resistant to fisheries collapses because of their early age at maturity, pelagic lifestyle (reducing the risk of habitat destruction) and their absence in the bycatch of other species [36]. Nevertheless, there are many examples of pelagic species that were thought to be resilient against population collapses, yet collapsed under excessive fishing pressure. Among these examples are clupeids like the Pacific sardine (*Sardinops sagax*) [37, 38], and the Atlantic herring (*Clupea harengus*) [39].

Previous attempts to reveal the population genetic structure of *S. tanganicae* and *L. miodon* are scarce*.* In *S. tanganicae,* the only genetic study conducted so far suggested a single panmictic stock [40], while in *L. miodon*, no clear large-scale geographic structure could be identified [41]. However, the genetic markers used in these studies (RAPD markers in *S. tanganicae*; allozyme markers and mtDNA Restriction Fragment Length Polymorphism (RFLP) of the ND 5/6 gene in *L. miodon*), may lack the sensitivity to detect genetic structure in highly dispersive organisms. Recent developments in sequencing technologies, such as Restriction site Associated DNA markers (RAD sequencing) allow to infer population structure based on numerous single nucleotide polymorphisms (SNPs) [42]. The accuracy of RAD sequencing in detecting low levels of genetic differentiation therefore exceeds the accuracy of molecular techniques based on other marker types, even at small sample sizes [40, 43]. Although commonly used to detect population structure in pelagic marine species, RAD sequencing has less often been used in pelagic freshwater species.

In this study, we combine an analysis of mitochondrial DNA (mtDNA) haplotypes with a genomic analysis of nuclear DNA to assess population structure of *S. tanganicae* along a north-south axis in LT. A heterogeneous population genetic structure is possible for three reasons. First, the distance between the northern and southern end of the lake is large, compared to the assumed migration distances of this species, so levels of mixing might decrease with distance (hypothesis of isolation by distance). Second, there are limnological differences between the North and the South of the lake, to which *S. tanganicae* might have distinct adaptations (hypothesis of adaptive evolution). Since the eggs slowly sink to a depth of 150 m in the South [20], and need to hatch before reaching the anoxic zone, we assume this leaves less time for the eggs in the North to develop. The inclination of the thermocline could have an effect on larval development and productivity. Finally, fluctuations in the lake water level stands [41] have affected connectivity in the lake. Lower connectivity limits migration, which could lead to population structuring (hypothesis of historical subdivision). Alternatively, since migration distances are not exactly known and these sardines are highly mobile and may have large effective population size, the species could be panmictic across the entire north-south axis. This panmixia would fit with observations in other sardines and anchovies, which often show low population differentiation [44].

## Material and methods

### Sampling and DNA extraction

**Fig. 1:**
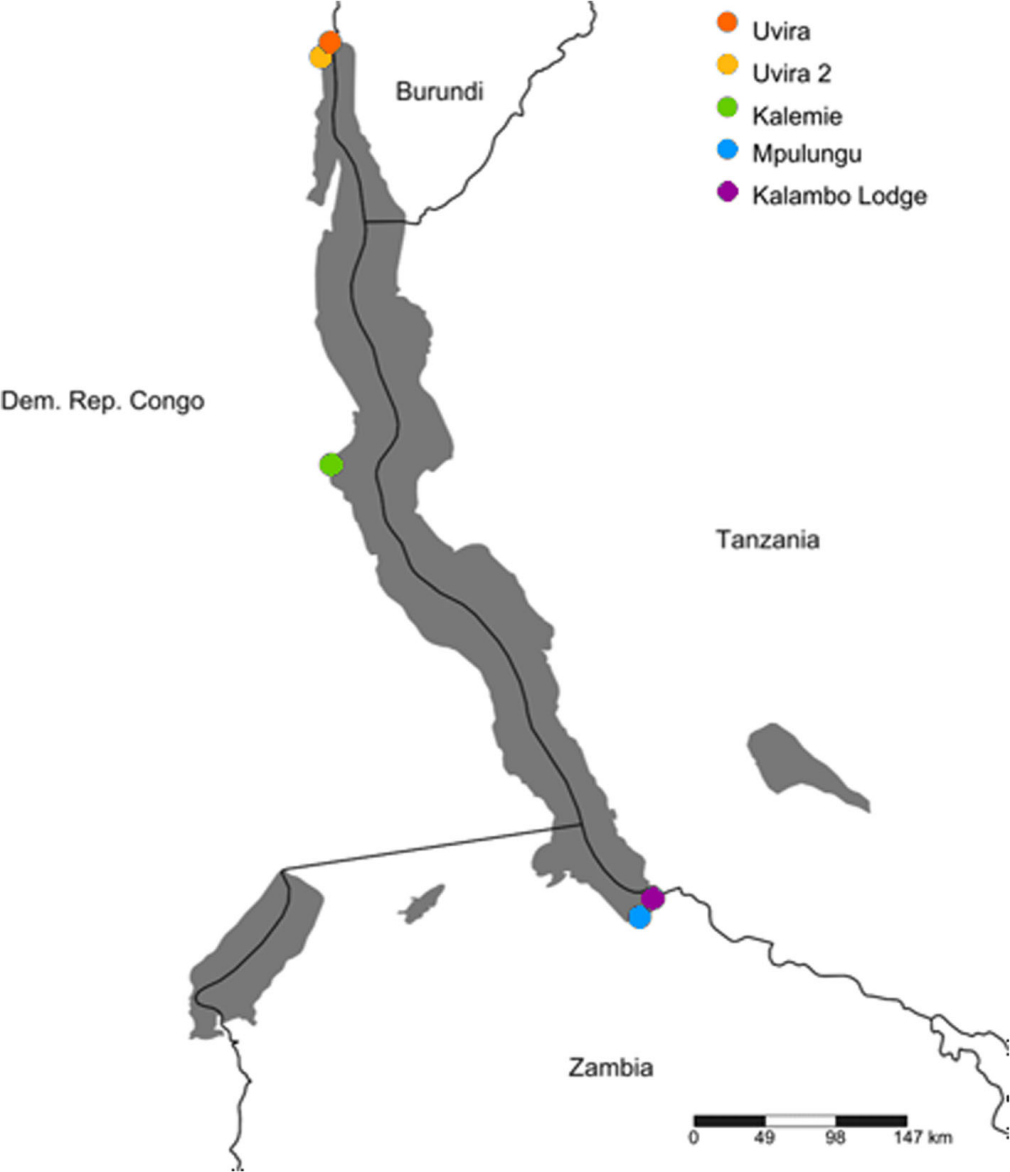
Map of Lake Tanganyika with sampling sites for *Stolothrissa tanganicae.* Uvira and Uvira2 are located in the northern subbasin, Kalemie in the central subbasin and Mpulungu and Kalambo Lodge are in the southern subbasin. Map made with Simple Mapper (http://research.amnh.org/pbi/maps/)

We selected five sampling sites along a latitudinal gradient covering the three subbasins of LT (Fig. 1). Two sites were selected in the northern basin (Uvira and Uvira 2), one in the central basin (Kalemie), and two in the southern basin (Mpulungu and Kalambo Lodge) (Table 1). This allowed us to evaluate population structure at the level of the entire lake, as well as among nearby sampling sites in the North and South of the lake. All samples were bought in the morning between August 11th and 20th 2016 (Table 1) from local fishermen who operated in a small range around the landing site. Since fishermen do not recast nets after they have been filled up by a passing school, all individuals within a sample belonged to the same school. To minimize the probability that a migrating school was sampled twice, the fish were bought on the same day for the two locations in the North, and on consecutive days for the two locations in the South (Table 1). For each sample, a finclip was stored in 99% ethanol. All individuals where measured and 32 were sexed of which 4 were male, 12 were female and 16 were not mature thus sex could not be identified. In total 96 individuals were used for the analysis of mitochondrial data and for the RAD library construction. DNA was extracted from finclips, using the NucleoSpin Tissue kit (Macherey-Nagel GmBH) according to the manufacturer’s instructions.

**Table 1:** Sampling information on *Stolothrissa tanganicae*

Site	n	Subbasin	Date	Longitude	Latitude
Uvira	16	northern	11/08/2016	−3.333539	29.189359
Uvira2	16	northern	11/08/2016	−3.395340	29.162933
Kalemie	32	central	12/08/2016	−5.947490	29.196633
Mpulungu	16	southern	19/08/2016	−8.762340	31.110506
Kalambo Lodge	16	southern	20/08/2016	−8.653927	31.195447

*Sample size (n), subbasin, date of sampling and coordinates for the five sampling site. Sites represent the landing sites where fresh fish were purchased*

### Mitochondrial sequence data

The mitochondrial cytochrome *c* oxidase subunit I (COI) gene was amplified using the universal primer combination HCO2198 (5’-TAAACTTCAGGGTGACCAAAAAATCA-3′) and LCO1490 (5’-GGTCAACAAATCATAAAGATATTGG-3′) [45]. The PCR mix consisted of 1 μL of template DNA, 2.5 μL PCR buffer, 0.75 μL Platinum MgCl_2_ (50 mM), 0.5 μL of dNTPs (10 mM), 1 μL of both primers (10 μM), 0.15 μL Platinum *Taq* polymerase (5 units/μL) and 18.1 μL of milli-Q water, totaling 25 μL. The PCR cycling profile consisted of 3 min at 94 °C, followed by 35 cycles at 94 °C for 45 s, 52 °C for 40 s, 72 °C for 90 s, 10 min at 72 °C and cooling to 4 °C. PCR products were purified by means of GFX purification columns (GE Healthcare, Chicago, IL, USA), subjected to sequencing reactions using the BigDye v3.1 cycle sequencing kit (Applied Biosystems, Foster City, CA, USA) and sequenced using the LCO1490 primer, with an ABI Prism 3100 Genetic Analyzer (Applied Biosystems). Sequence quality was verified with Geneious v11 [46] and MEGA v7.0 [47] by checking each SNP for base quality, assuming a reading error if a SNP is rare and quality is low. We checked for mutations recorded on the second position in a codon, which did not occur. Sequences were aligned with MUSCLE [48] using the default settings (Gap penalties: open = − 400; extend = 0, clustering method UPGMB, λ = 24). Before analyses, primers were trimmed out and sequences translated into amino acids to check for the absence of internal stop codons. Given the absence of gaps, the alignment was straightforward. The mitochondrial sequence data were used (a) to double-check the morphological identification of voucher specimens via DNA barcoding (data not shown) and (b) to assess possible genetic structure across individuals from different sampling sites. For this, a Median Joining Network [49] was made with PopART 1.7 [50], with ε = 0. Differentiation among individuals from the different sampling sites was estimated by global F_ST_ and pairwise F_ST_ between sampling sites in the diveRsity package [51] in R, using 100 bootstraps to calculate bias corrected 95% confidence intervals. We calculated number of haplotypes and Tajima’s D statistic, using DnaSP v6 [52].

### RAD library preparation

Six RAD libraries, each including 16 individually indexed specimens, were prepared according to the protocol described in Baird et al. [53] and Etter et al. [54]. Individual DNA samples were digested using restriction enzyme *SbfI-HF* (NEB, cut site 5’-CCTGCA^GG-3′). In silico digestion of the genome of the related Atlantic herring (*Clupea harengus*) [55] revealed 21,544 RAD loci with SbfI. Samples were individually barcoded with P1 adapters ligated to the fragment’s overhanging end. The RAD libraries were sheared to a size of 350 base pairs (bp) and the fragments between 200 and 700 bp selected by gel size selection. A second, library-specific barcoded adapter (P2), was ligated to the DNA fragments for identification of the samples. RAD libraries were sequenced 101 bp paired-end on an Illumina HiSeq1500 platform at the Medical Centre for Genetics of the University of Antwerp, Belgium.

### Processing of RAD data

Overall read quality was assessed using the FastQC software v0.11.5 [56]. Raw sequence data was demultiplexed using the *process_radtags* module in Stacks v1.46 [57, 58], while reads characterized by ambiguous barcodes, ambiguous cut sites or low quality scores were discarded. PCR duplicates were removed via the *clone_filter* module and SNPs were called using the *denovo_map* pipeline, both implemented in Stacks. We screened a range of parameter combinations and selected a minimum coverage of ten reads per stack (m = 10) and a maximum number of five base pair differences between stacks within (M = 5) and between (*n* = 5) individuals. This parameter setting allowed us to retain a sufficient number of orthologues at a considerable depth. Individuals with insufficient raw reads (< 0.8 million), a high proportion of missing data (> 50%) and low depth (< 9.7) were removed. A final round of filtering was performed using VCFtools v0.1.14 [59] in order to discard sites characterized by heterozygosity excess (*p*-value < 0.01), a minimum allele frequency of less than 0.05, and more than 20% of missing data.

### Neutral population structure

Genetic variation of each sample was assessed by expected and observed heterozygosity and allelic richness, using the diveRsity v1.9.90 package in R v3.4.1. Using the same package, estimates of global F_ST_ were calculated, for two geographic scales, lake-wide and between nearby locations. To make lake-wide comparisons, we pooled the two northern (Uvira + Uvira2) and the two southern (Mpulungu + Kalambo Lodge) sampling sites. Pairwise F_ST_ was calculated across the sampling sites, with 95% confidence intervals based on 100 bootstrap iterations over loci.

Population structure was inspected with the R package ADEGENET v2.1.0 [60] to perform a non-centered, non-scaled Principal Coordinates Analysis (PCoA) based on Euclidean distances between specimens. Missing data in this analysis were replaced by the mean allele frequencies. In addition, we performed a Discriminant Analysis of Principal Components (DAPC) [61] with default settings. DAPC reduces the variation within the sampling sites, while maximizing the variation between them. As the amount in explained variance showed a continuous gradual decline with most important PCs, no optimal cutoff of number of PCs could be identified. Therefore, the DAPC was based on 28 PCs, the largest number of informative PCs [60].

Population structure was assessed using an MCMC method to infer recent shared ancestry based on patterns of genomic similarity implemented in fineRADstructure [62], which is a modification of fineSTRUCTURE [63] for RAD data. As this analysis showed to be highly sensitive to missing data, we retained only SNPs scored in more than 90% of all individuals. The RAD tags were ordered according to linkage disequilibrium with the sampleLD.R script provided in fineRADstructre. Subsequently, the co-ancestry matrix was calculated and used to identify populations by a clustering algorithm. This approach is robust for missing RAD alleles and is sensitive to subtle population structure. The MCMC chain ran with a burnin of 100,000, 100,000 iterations and a thinning interval of 1000. We further explored whether genetic similarity between individuals decreased with geographical distance by conducting a spatial autocorrelation analysis over the five sampling sites in GenAlEx v6.501 [64–66]. Correlation coefficients between individuals were depicted as a function of increasing inter-individual geographical distance and confidence intervals based on 1000 bootstraps.

### Genome scan of outlier loci

Putative signatures of natural selection were assessed using three different approaches to detect outlier loci. First, we assessed the distribution of the global F_ST_ values among loci, at the lake-wide scale and between all five of the locations, to identify possible candidates for outliers, using the diveRsity v1.9.90 package of R. Secondly, we performed a Bayesian outlier detection method in BayeScan v2.1 [67] which incorporates locus- and population-specific F_ST_ effects [67–69]. For each level, three replicate runs were executed with default parameter settings. False discovery rate (FDR) threshold was 0.05, and only loci consistently identified as outliers in each of three independent runs were considered as true outliers. Finally, we assessed the possible occurrence of adaptation along the latitudinal gradient. We applied an individual-based latent fixed mixed model (LFMM) in which SNP frequencies were associated to latitudinal variation, while accounting for neutral population structure [70]. The number of latent factors was set to one. We ran the model ten times, using 20,000 sweeps for burn-in and 40,000 additional sweeps as run-length and calculated the median Z-value of all replicated runs for each locus separately. We applied a correction by dividing the raw *p*-value by a genomic inflation factor, corresponding to the median of the square z-value divided by the median of the chi-square distribution [71]. To correct for the multiple tests, SNPs that were considered as non-neutral, were characterized by a q-value of 0.05 or less [71]. LFMM analyses were performed using the LEA package in R [72].

## Results

### Phylogeography based on mitochondrial sequence data

**Fig. 2:**
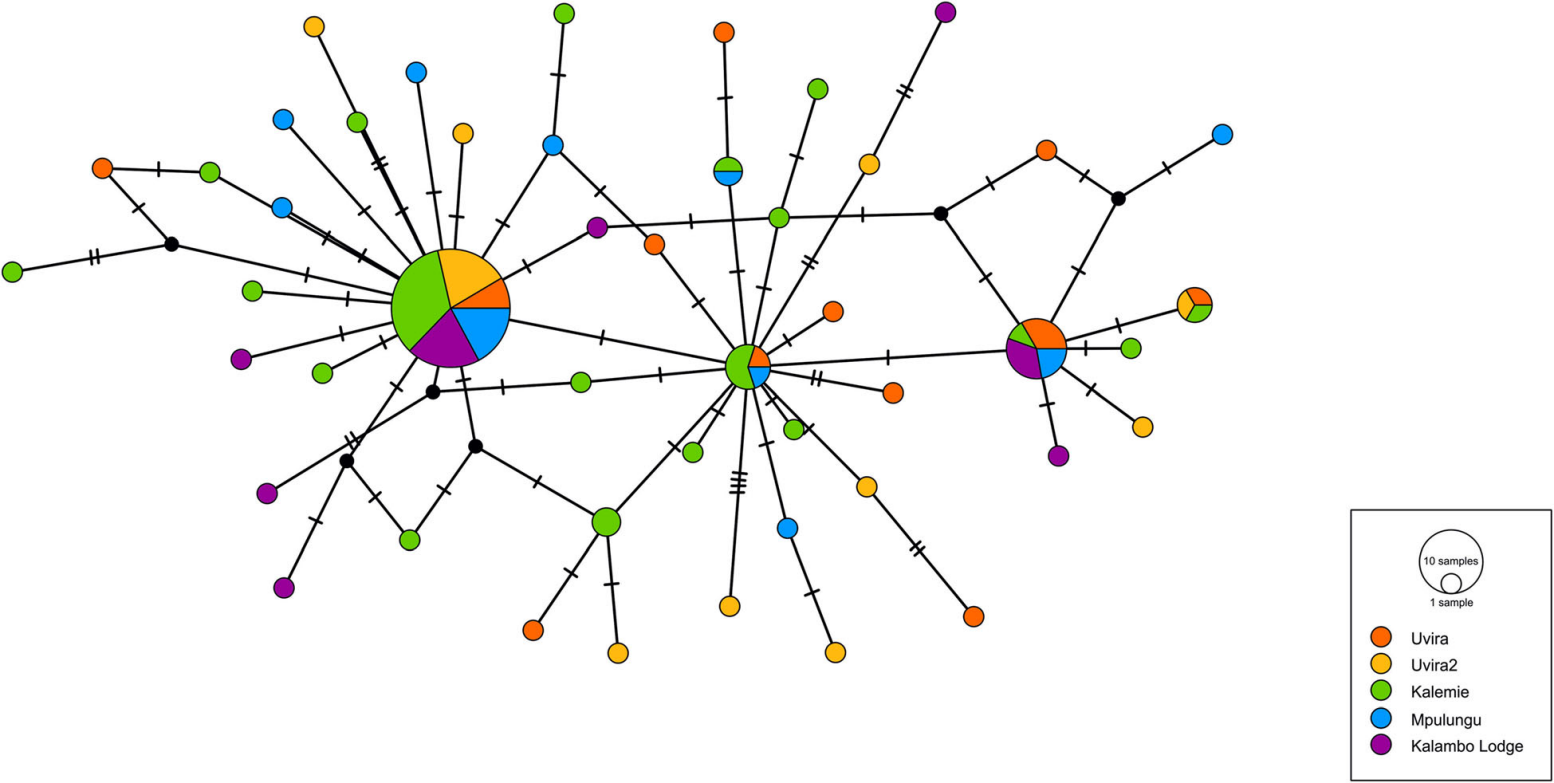
Haplotype network of COI sequences of *Stolothrissa tanganicae* (*n* = 96). Median Joining Network (ε = 0) created in PopART v1.7. Each circle represents a haplotype, the size of circles corresponds to the number of individuals with the haplotype. Colors indicate sampling sites. Bars indicate the number of mutations between two haplotypes. Small black circles indicate hypothetical haplotypes, predicted by the model. Uvira and Uvira 2 are in the northern basin, Kalemie is in the central basin and Mpulungu and Kalambo Lodge are in the southern basin

The Median Joining Network based on mitochondrial COI fragments of a length of 643 bp, does not suggest separation either between the five sampling sites, or between the three subbasins (Fig. 2). The global F_ST_ value between sampling sites is 0.0026. Pairwise F_ST_ values (Table 2) do not significantly differ from zero. Values range from -0.027 (95% CI: - 0.072, 0.0641) (Mpulungu – Uvira 2) to 0.0327 (95% CI: -0.0358, 0.1498) (Kalemie – Uvira). In these 96 samples, there are 47 different haplotypes and Tajima’s D is significantly negative (D = -2.414, *p* < 0.01).

**Table 2:** Pairwise genetic differentiation (F_ST_) between sampling sites of *Stolothrissa tanganicae*

F_ST_	Uvira	Uvira 2	Kalemie	Mpulungu	Kalambo lodge
Uvira		0.0077 [− 0.0621, 0.1302]	0.0316 [− 0.0368, 0.1496]	0.0206 [− 0.0579, 0.1609]	0.03267 [− 0.0358, 0.1498]
Uvira 2	0.0044 [0.0023, 0.0066]		−0.0200 [− 0.0650, 0.0551]	− 0.0275 [− 0.0720, 0.0641]	−0.0247 [− 0.0787, 0.0803]
Kalemie	0.0250 [0.0215, 0.0281]	0.0045 [0.0029,0.0066]		−0.0136[− 0.051, 0.0463]	−0.0035[− 0.0485, 0.0524]
Mpulungu	0.0166 [0.0140, 0.0194]	0.0017 [-0.0001, 0.0041]	0.0014 [-0.0005, 0.0030]		−0.0219 [− 0.0782, 0.0584]
Kalambo Lodge	-0.0012 [-0.0028, 0.0001]	-0.0005 [-0.0024, 0.0010]	0.0100 [0.0085, 0.0010]	0.0031 [0.0014, 0.0051]	

*Values below the diagonal are from the nuclear DNA, above the diagonal from mitochondrial data. The values in brackets represent 95% confidence intervals based on 100 bootstraps over loci*

### Quality of RAD genotyping

Due to a low number of reads (< 0.8 million), a high percentage of missing reads (> 50%) and low depth (< 9.7), 12 individuals were discarded. Two individuals from the Mpulungu sampling site were very similar, indicating possible contamination. To solve this, one of these individuals was removed. This resulted in 83 retained individuals, at least 15 per sampling site, with the number of reads per specimen ranging from 0.9 to 3.7 million (average per specimen = 1.96 million). Filtering produced a final dataset containing 3504 SNPs distributed across these 83 individuals, with a mean depth per individual of 29.66 (minimum of 9.7 and maximum of 57.9) and a mean missing per individual of 12% (minimum of 0.007% and maximum of 48%). Detailed information on missing data per individual can be found in Additional file 1.

### Nuclear genetic diversity and neutral population structure

Observed and expected heterozygosity values were similar among the sampling sites, with expected heterozygosity ranging from 0.2088 (Kalemie) to 0.2605 (Uvira) and observed heterozygosity ranging from 0.1920 (Kalemie) to 0.2619 (Uvira) (Table 3). Allelic richness across the different sampling sites ranged from 1.7831 (Kalemie) to 1.9047 (Mpulungu) (Table 3).

**Table 3:** Nuclear genetic diversity of *Stolothrissa tanganicae* by sampling site

	Sample size	H_e_ (mean ± SE)	H_o_ (mean ± SE)	AR (mean ± SE)
Uvira	15	0.2605 ± 0.0024	0.2619 ± 0.0029	1.8716 ± 0.0041
Uvira2	15	0.2301 ± 0.0024	0.2213 ± 0.0025	1.8401 ± 0.0044
Kalemie	22	0.2088 ± 0.0025	0.1920 ± 0.0026	1.7831 ± 0.0046
Mpulungu	15	0.2529 ± 0.0023	0.2565 ± 0.0026	1.9047 ± 0.0033
Kalambo Lodge	16	0.2232 ± 0.0025	0.2181 ± 0.0027	1.8408 ± 0.0040

*Expected and observed heterozygosity (H*_*e*_ *and H*_*o*_ *) and allelic richness (AR) by sampling site. Sample size is the number of individuals used for the analysis of the RADseq data, after exclusion of low quality samples. SE: standard error*

Genetic differentiation estimated by global F_ST_ was relatively low, but significantly different from zero, with a F_ST_ value of 0.0068 (95% CI: 0.0057–0.0079) between sampling sites and a F_ST_ value of 0.0054 (95% CI: 0.0046–0.0066) between the northern, central and southern basin. Similarly, pairwise F_ST_ values between sampling sites are low, ranging from -0.0012 (95% CI: -0.002–0.0001) (Kalambo Lodge – Uvira) to 0.0250 (95% CI: 0.0215–0.0281) (Kalemie – Uvira) (Table 2).

**Fig. 3:**
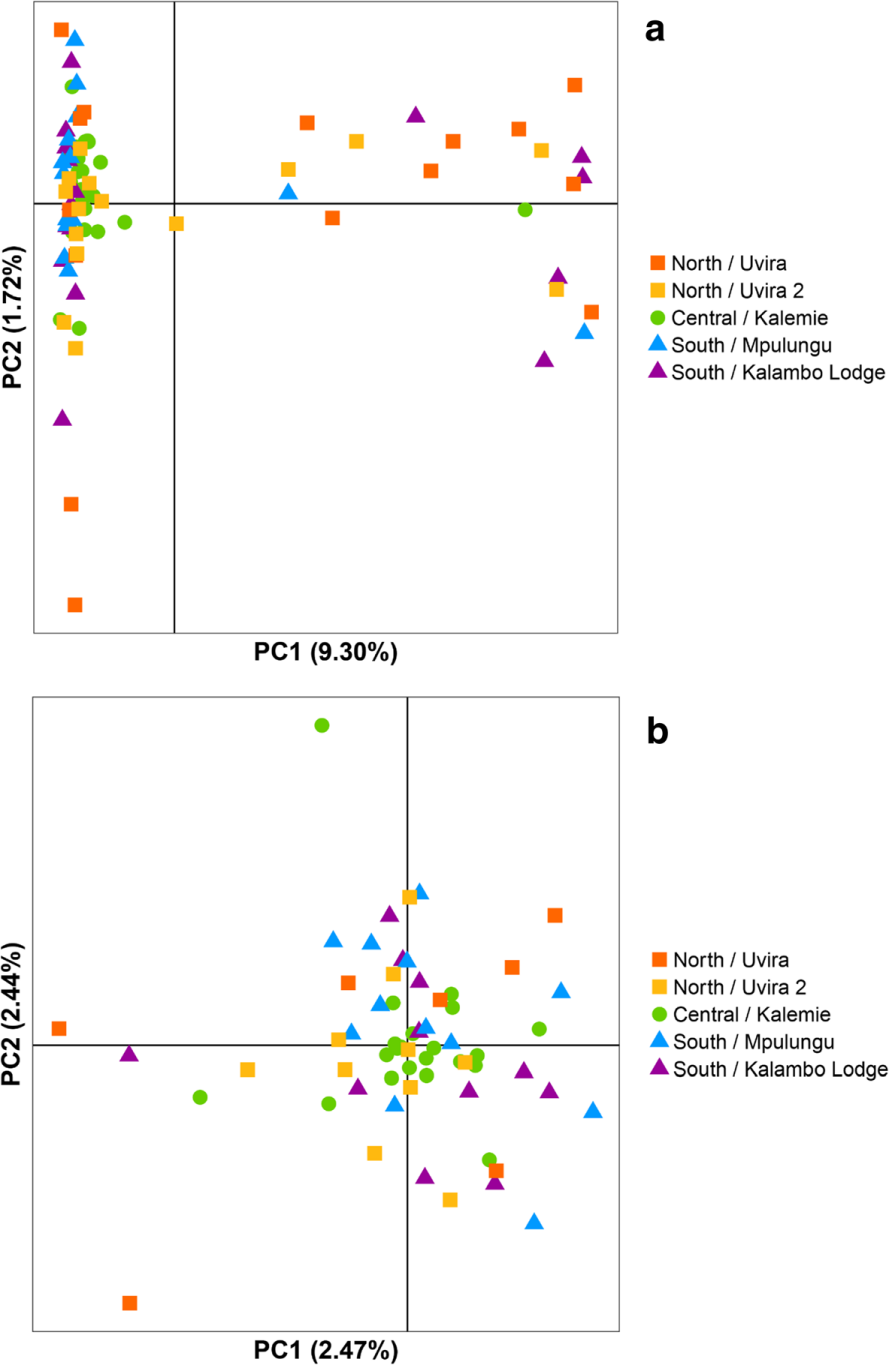
PCoA based on kinship of nuclear DNA. Each dot represents one *S. tanganicae* individual. Dots that are closer together have more similar genotypes. Colors represent the five sampling sites. **a**. all individuals, PC1 explains 9.30% of the variation and PC2 1.72% of the variation. **b**. Plot with only the individuals from the larger cluster, PC1 2.47% explains of the variation and PC2 explains 2.44% of the variation. Made with ADEGENET v2.1.0 package in R

**Fig. 4:**
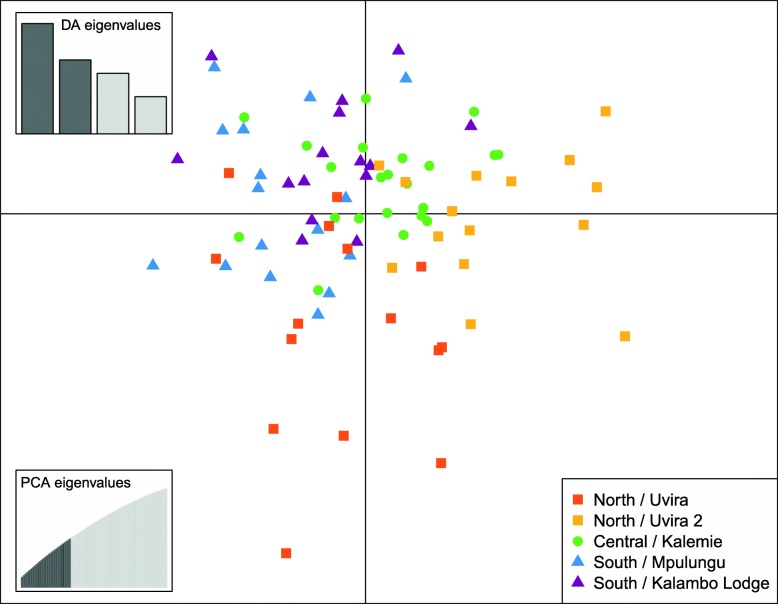
Discriminant analysis of principal components (DAPC) with a priori grouping corresponding to the sampling sites of *Stolothrissa tanganicae.* Scatterplot of DAPC data based on nuclear DNA

**Fig. 5:**
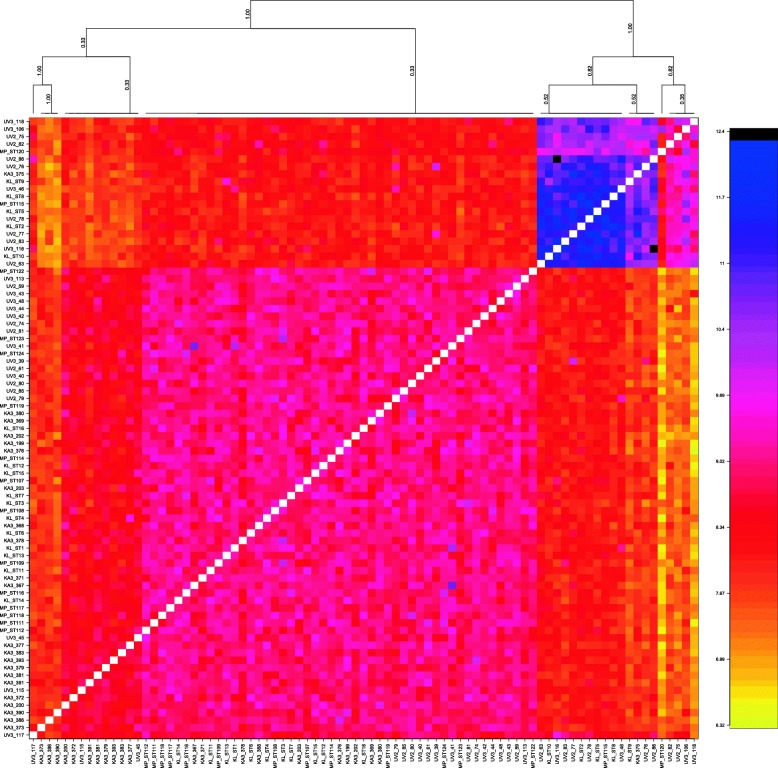
FineRADstructure analysis for visualization of patterns of haplotype similarity: co-ancestry matrix based on a reduced dataset of 1255 SNPs. Colors indicate scale of relatedness between individuals, with yellow being low relatedness and blue/black indicating high relatedness. No structuring per sampling site is visible. A cluster of individuals is apparent in the upper right of the graph. These individuals correspond to the individuals that score high on the first axis of the PCoA plot, and are spread over the different sampling sites. Made with the fineRADstructure software [62]

The PCoA revealed no clustering based on the geographic origin of the samples (Fig. 3a). Individuals are separated on PC1 (9.30% explained variation) in one large cluster of 63 individuals and one smaller cluster of 20 individuals, regardless of the sampling site. PC2 and PC3 explained 1.72 and 1.71% of the variation respectively. PCoA was repeated with only the individuals in the larger cluster, to check if there is no hidden structure (Fig. 3b). Here, PC1 explains 2.47% of the variation and PC2 explains 2.44%. F_ST_ between the large and smaller cluster is significantly different from zero: 0.1338 [0.1239,0.1445]. The DAPC analysis shows no obvious pattern of genetic structuring across sampling sites, although some degree of separation on the diagonal is visible, with the samples from the central basin placed between those from the North and those from the South (Fig. 4, Additional file 2). For the visualization of patterns of haplotype similarity with fineRADstructure (Fig. 5), a reduced dataset of 1255 SNPs was used, to correct for effects of unevenly distributed levels of missing data. The structure provided by the fineRADstructure analysis corroborated with the results of the PCoA analysis, placing the same individuals in the same two clusters. These two groups are irrespective of sex or sampling site.

**Fig. 6:**
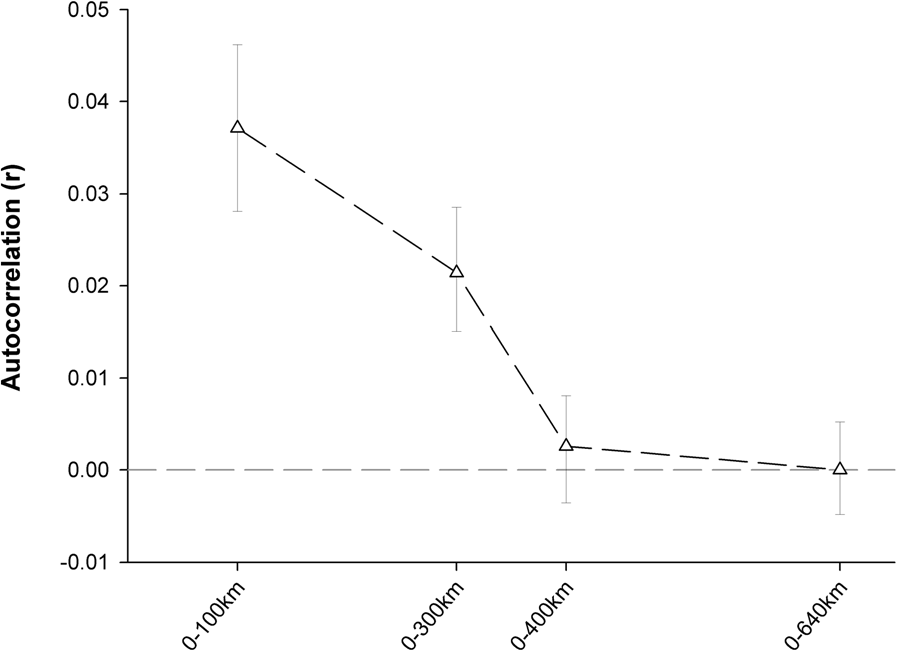
Autocorrelation (r) showing genetic similarity over geographical distance. Error bars bound the 95% confidence interval as determined by bootstrap resampling. Over a distance of 400 km, 95% CI include zero, showing that random processes like stochastic drift overcome the homogenizing effect of gene flow. Analysis done in GenAlEx v6.501 [66]

Autocorrelation analysis shows a low but significant level of genetic structuring in the five sampling sites along a north-south axis of LT, indicating a difference in populations in the North compared to the South. At a distance of 400 km, random processes like stochastic drift seem to overcome the homogenizing effect of gene flow (Fig. 6).

### Outlier loci

Patterns in global F_ST_ at each SNP are concordant with previous results as the majority of F_ST_ values are clustered around zero, indicating low levels of genetic structuring (Additional file 3). Only 32 SNPs are characterized with a F_ST_ higher than 0.1 according to sampling site and 12 SNPs according to subbasin. The highest F_ST_ value is 0.21. None of these are identified as significant outliers by BayeScan (Additional file 4) or LFMM (Additional file 5) at a FDR threshold of 0.05.

## Discussion

### Population structure of *Stolothrissa tanganicae*

The population structure of *Stolothrissa tanganicae* was explored over five sampling sites in the three subbasins of LT using mitochondrial COI sequences and RAD sequencing data, to verify the existence of biologically meaningful management units. This species showed for both marker types a very weak genetic structure, suggesting a near-panmictic population. For both markers, the difference between samples from the different subbasins is not larger than the difference within subbasins. This pattern is obvious in both the PCoA and fineRADstructure analysis of the RAD data and the haplotype network based on the mitochondrial DNA. The PCoA plot (Fig. 3) and the Median Joining Network (Fig. 2), show no genetic structuring according to sampling site or subbasin. Autocorrelation analysis revealed that there is a limitation to long-distance migration, as at a distance of 400 km, a decline in gene flow becomes apparent. The high number of different mitochondrial haplotypes (47), suggests many different maternal lineages.

The diverse set of lineages and the overall weak genetic structure confirm the conclusions of a previous population genetic study on *S. tanganicae*, which suggested a single panmictic stock [40]. However, this study was based on random amplified polymorphic DNA (RAPD) markers. RAPD markers are often difficult to interpret, and the results are not always reproducible. Our confirmation of the results based on a large set of high-quality SNPs represents an important benchmark, and indicates that *S. tanganicae* has been near-panmictic since the 1990s. No other population genetic studies on *S. tanganicae* are available, but a study by Sako et al. [43] revealed significant differences in otolith chemistry between populations from the northern and southern basin. This difference suggests that populations from the North and South of the lake spend most of their lifetime in different environments and implies that long-distance migrations must be rare. This seems to contradict the genetic patterns. However, a few migrants per generation are usually sufficient to maintain a near-panmictic population at the level of the entire lake.

Some of our analyses suggested the existence of two separate groups, independent of geographical origin. This is apparent in the PCoA plot (Fig. 3a), where we found a separation along the first axis. FineRADstructure analysis revealed the same two clusters. It is unclear what difference there is between these two groups, which differ in size. Missing data were equally distributed among the groups, so this is not the origin of the separation. The two groups could point to the two different sexes, yet for the 16 individuals that have been sexed in this study, male and female individuals show up in both groups in both analysis. Separate groups may as well arise because of a difference in spawning times. There are currently no indications for this in *S. tanganicae*, but it has been shown for Atlantic herring where spring-spawning and autumn spawning individuals were genetically differentiated [73]. Another possibility is that *S. tanganicae* frequently hybridizes with the other endemic clupeid, *L. miodon,* since F_ST_ between both groups is very large (F_ST_ = 0.1338 [0.1239,0.1445]). A larger sample size, as well as individuals of both species, are required to test the identity of the two separate groups. Individuals from the two groups have been found in all the sampling sites, so they do not alter the conclusions of the various analyses in this study.

### The lakescape of pelagic fish

It is worthwhile to speculate what may cause the weak geographical population genetic structure of *Stolothrissa tanganicae*. At the start of this study, we hypothesized that population structure could arise due to isolation by distance, adaptive evolution, or the distinct history of the subbasins. We also considered the possibility of a homogeneous population because of large effective population sizes and high mobility of the species and the long period during which obvious migration barriers were absent. Our data did not show genetic differentiation between the different sampling locations over a north-south axis of the lake.

First, the data revealed a very weak pattern of isolation by distance, which was detected with the autocorrelation analyses. In 1970, Coulter stated, based on his observations in the northern and southern basins, that there were no reports of large clupeid migrations, and that there was no reason to assume there were any [25]. Yet, as stated above, some migration either individually or in schools may cause sufficient gene flow to keep the population structure near-panmictic. Similar to the marine environment, the pelagic zone of LT does not contain many barriers for migration. The frequent algal blooms in LT attract zooplankton, which in turn attracts the sprats. These algal blooms occur in the South of the lake in May–June, due to upwelling of nutrient rich water caused by tilting of the epilimnion because of strong south-east winds. After the winds cease around September, currents reverse and an algal bloom occurs in the North in October–November [16]. Migrations follow these blooms, as indicated by a positive correlation between *S. tanganicae* abundance and measures of chlorophyll *a* [23]. Catch statistics indicate a peak in *S. tanganicae* catches during phytoplankton blooms in the North [23, 74] and the South [75] of the lake. These seasonal migrations may contribute to the mixing of populations.

We found no traces of local adaptation to different conditions in the North and the South of LT. The number of loci in this study may have been too low to detect genomic regions involved in adaptive processes. There are some limnological differences along a north-south axis that could trigger local adaptation. For instance, the timing of major spawning events in *S. tanganicae* differ across the lake [21, 25, 28], but it is unknown if this difference in spawning time is an adaptive trait or linked to phenotypic plasticity in response to the timing of the plankton blooms [28] and depth of the oxygenated layer. Little is known about spawning areas and mating behaviour of the sprat. There is little information on how the eggs are fertilized and deposited and about dispersal of eggs, both possible facilitators of population mixing, as has been shown for marine species [76]. Expanding this limited knowledge is needed for good monitoring and conservation of the stock, and could help in explaining why the population remains homogeneous.

Our results do not show signatures of a population that differentiated because of historical barriers, which would have caused greater differences in genotypes between our samples. At times of extreme low-stands of the water levels of LT, the lake would be divided into three separate lakes, according to the three subbasins. It is assumed that the differentiation in cichlids was triggered by this isolation [77–79]. It is unclear if *S. tanganicae* also differentiated into different populations in the isolated subbasins as a result of low water levels. This lack of observed differentiation could be due to the pelagic life style of the sprat, enabling dispersal throughout the lake, similar to the benthopelagic Lake Tanganyika’s giant cichlid (*Boulengerochromis microlepis*) [80] and two eupelagic *Bathybates* species (*B. fasciatus* and *B. leo*) [81] whose populations also do not show any phylogeographic structure.

A possible explanation for the homogeneous structure found here for *S. tanganicae* is that these populations could have passed through a bottleneck and quickly expanded again. This assumption is supported by a significant negative value of Tajima’s D statistic, showing that observed heterozygosity is lower than expected heterozygosity due to inbreeding. Clupeids are known to have highly fluctuating population sizes, with large declines in numbers and fast expansions [82], leading to traceable bottlenecks [44]. Fishing pressure, poor recruitment or limited food availability could have significantly reduced the number of remaining sprats. Lake Tanganyika sprat is an r-selected species [83] with a short lifespan, many offspring and reaching an age of maturity within a few months [84]. Furthermore, schooling reduces the effort to find a mate. This makes *S. tanganicae* excellently equipped for rapid population expansions [20].

Just like *S. tanganicae* in this study, sardines worldwide, often assessed over greater geographic distances, show below-average levels of population differentiation in comparison to other marine fishes. This is generally explained by their pelagic lifestyle, limited proportion of the population that contributes to the next generation, overharvesting and population bottlenecks [44, 85, 86]. In some cases, population genetic structure was detected [87], for example in the presence of physical barriers such as ocean currents [88] or over large geographical distances [89]. In other cases, subtle levels of ecological adaptation have been detected, for example between Atlantic herring (*Clupea harengus*) from the North Sea [90] and the Baltic Sea [91].

### Implications for fisheries management and future research

The weak genetic structure in *S. tanganicae* over a north-south axis of LT, emphasises the need for integrated management of the entire stock. On the one hand, a single homogeneous stock might be easier to manage, since local extinctions can be countered by migrations from other populations. The adaptive potential and chance of survival of a metapopulation is bigger than that of an isolated subpopulation. On the other hand, managing such a homogeneous population has its own difficulties: Lake Tanganyika is bordered by four countries, each with its own legislation, law enforcement and economic reality. As the geographically unstructured sprat stocks do not correspond to international borders, each local management regime influences the stock available to the neighbouring countries. Our findings also underpin the importance of locating and protecting the spawning areas of *S. tanganicae*, since degradation of a spawning area could impact the stock in a wider area. Illegal fishing of clupeid fry in the spawning areas forms a huge burden on the stocks [92, 93]. It is also important to have more knowledge on which parts of the lake serve as sources and which as sinks for the *S. tanganicae* population. This information is vital to delineate spawning areas and source populations as protected areas.

Future research on the pelagic species in Lake Tanganyika remains necessary to provide information for management and conservation. More information on migrations of these pelagic clupeids would be beneficial for more directed management. The availability of a reference genome would be a step towards interpretation of adaptive traits if outlier SNPs would be detected. It will also be vital towards discovering genomic signatures of overfishing. There is also a need to look at the population structure of the two other major fisheries target species in Lake Tanganyika, *L. miodon* and *L. stappersii*. They both have a more littoral lifestyle than *S. tanganicae* [21], hence their populations might be more structured. Also, *L. stappersii* has a very different life history than the clupeids: these predators are bigger and live longer, which might affect their population structuring. This type of research can be useful in many other systems. It can be expanded to pelagic fish of the other African Great Lakes, and beyond. There are many lake ecosystems where a small, fast growing, pelagic fish species forms the link between zooplankton and piscivorous animals, just like the clupeids of Lake Tanganyika. Many of these systems would benefit from having information about the population structure of their pelagic fisheries targets. In some of these lakes, for example Lake Victoria, the pelagic fishes are becoming more important in the ecosystem due to overfishing of the larger fish species.

## Conclusion

Our study confirms previous findings on the population structure of *S. tanganicae* in Lake Tanganyika. A near-panmictic population structure was detected over a north-south axis of the lake, with slightly increasing genetic distance over increasing geographical distance. This homogeneity in the stock of one of the major fisheries target species in LT underscores the need for integrated stock management between the four nations bordering Lake Tanganyika.

## Additional files

Additional file 1: Sequencing quality and information on missing data per individual. Table shows the sampling site, individual ID, number of SNPs, number of missing SNPs, frequency of missing SNPs, mean read depth and number of raw reads per individual. (PDF 370 kb)

Additional file 2: Density plot of DAPC. Densities of individuals on the first discriminant function of the DAPC shown in Fig. 4. (PDF 28 kb)

Additional file 3: Frequency distribution of global F_ST_ of *Stolothrissa tanganicae* per SNP. Grouping by sampling site and subbasin. (PDF 5 kb)

Additional file 4: Outlier analysis based on BayeScan v2.1. A. F_ST_-Log10 posterior probability for two levels (sampling site and subbasin) for each of the three replicates (R1, R2, R3). B. Q value for grouping according sampling site and subbasin with each of the three replicates (R1, R2, R3). (PDF 169 kb)

Additional file 5: Individual-based latent fixed mixed model (LFMM) analysis. Distribution of adjusted *p*-values, corrected with the genomic inflation factor. Made with the LEA package in R. (PDF 4 kb)

## Abbreviations

bp: Base pairs
CI: Confidence interval
COI: Cytochrome *c* oxidase subunit I
FDR: False discovery rate
Kya: Thousand years ago
LFMM: Latent fixed mixed model
LT: Lake Tanganyika
mtDNA: Mitochondrial DNA
NGS: Next Generation Sequencing
RAD: Restriction site associated DNA
RFLP: Restriction fragment length polymorphism
SE: Standard error
SL: Standard length
SNP: Single nucleotide polymorphism
