# Supplementary material for: First genomic study on Lake Tanganyika sprat Stolothrissa tanganicae: a lack of population structure calls for integrated management of this important fisheries target species
Source: BMC Evol Biol. 2019 Jan 8;19:6. doi: 10.1186/s12862-018-1325-8 (PMC6323704; doi:10.1186/s12862-018-1325-8)
Supplement: Supplementary file 1 — Sequencing quality and information on missing data per individual. Table shows the sampling site, individual ID, number of SNPs, number of missing SNPs, frequency of missing SNPs, mean read depth and number of raw reads per individual. (PDF 370 kb) [file 12862_2018_1325_MOESM1_ESM.pdf]

Sampling site	Individual	Number of SNPs	Number of missing SNPs	Frequency missing	Mean depth	Raw reads
Kalemie	KA3_199	3504	97	0,03	35,09	2.146.802
Kalemie	KA3_200	3504	592	0,17	26,32	1.820.022
Kalemie	KA3_202	3504	467	0,13	24,34	1.869.078
Kalemie	KA3_203	3504	326	0,09	23,1	2.023.238
Kalemie	KA3_367	3504	257	0,07	23,76	1.798.612
Kalemie	KA3_368	3504	30	0,01	43,56	2.720.074
Kalemie	KA3_369	3504	378	0,11	27,42	2.058.304
Kalemie	KA3_371	3504	360	0,1	24,7	1.501.858
Kalemie	KA3_372	3504	797	0,23	19,53	1.326.124
Kalemie	KA3_373	3504	1656	0,47	13,3	883.788
Kalemie	KA3_375	3504	418	0,12	22,69	1.412.396
Kalemie	KA3_376	3504	43	0,01	37,28	2.105.898
Kalemie	KA3_377	3504	1002	0,29	15,79	1.836.456
Kalemie	KA3_378	3504	602	0,17	21,44	1.396.782
Kalemie	KA3_379	3504	667	0,19	19,78	2.361.746
Kalemie	KA3_380	3504	184	0,05	30,94	2.906.308
Kalemie	KA3_381	3504	676	0,19	23,72	1.663.752
Kalemie	KA3_383	3504	1008	0,29	14,49	1.093.970
Kalemie	KA3_386	3504	1176	0,34	16,27	1.317.082
Kalemie	KA3_390	3504	1632	0,47	9,73	936.940
Kalemie	KA3_391	3504	1062	0,3	16,46	1.629.154
Kalemie	KA3_393	3504	1071	0,31	15,37	1.418.702
Kalambo Lodge	KL_ST1	3504	111	0,03	39,98	2.280.154
Kalambo Lodge	KL_ST10	3504	118	0,03	39,46	2.347.430
Kalambo Lodge	KL_ST11	3504	55	0,02	48,19	3.202.962
Kalambo Lodge	KL_ST12	3504	24	0,01	50,97	3.107.996
Kalambo Lodge	KL_ST13	3504	94	0,03	38,09	2.628.646
Kalambo Lodge	KL_ST14	3504	88	0,03	39,5	2.496.334
Kalambo Lodge	KL_ST15	3504	94	0,03	34,72	2.360.362
Kalambo Lodge	KL_ST16	3504	101	0,03	32,39	2.025.520
Kalambo Lodge	KL_ST2	3504	74	0,02	49,84	3.006.300
Kalambo Lodge	KL_ST3	3504	92	0,03	57,95	3.651.424

Kalambo Lodge	KL_ST4	3504	70	0,02	38,68	2.318.954
Kalambo Lodge	KL_ST5	3504	136	0,04	37,49	2.290.728
Kalambo Lodge	KL_ST6	3504	36	0,01	40,18	2.413.374
Kalambo Lodge	KL_ST7	3504	94	0,03	30,99	1.937.878
Kalambo Lodge	KL_ST8	3504	203	0,06	33,75	2.173.122
Kalambo Lodge	KL_ST9	3504	786	0,22	16,98	1.243.716
Mpulungu	MP_ST107	3504	287	0,08	23,05	1.617.550
Mpulungu	MP_ST108	3504	192	0,05	30,84	2.074.750
Mpulungu	MP_ST109	3504	113	0,03	35,46	2.382.310
Mpulungu	MP_ST111	3504	178	0,05	27,64	1.799.564
Mpulungu	MP_ST112	3504	80	0,02	38,95	2.490.730
Mpulungu	MP_ST114	3504	207	0,06	30,88	1.783.014
Mpulungu	MP_ST115	3504	110	0,03	31,76	1.902.838
Mpulungu	MP_ST116	3504	62	0,02	41,2	2.390.546
Mpulungu	MP_ST117	3504	99	0,03	43,62	2.614.590
Mpulungu	MP_ST118	3504	71	0,02	35,99	2.128.834
Mpulungu	MP_ST119	3504	236	0,07	24,56	1.911.942
Mpulungu	MP_ST120	3504	1639	0,47	9,88	894.950
Mpulungu	MP_ST122	3504	117	0,03	33,81	2.114.302
Mpulungu	MP_ST123	3504	564	0,16	19,29	1.322.534
Mpulungu	MP_ST124	3504	95	0,03	31,54	1.960.586
Uvira	UV2_59	3504	68	0,02	45,83	2.866.482
Uvira	UV2_61	3504	50	0,01	37,99	2.494.088
Uvira	UV2_63	3504	68	0,02	36,54	2.261.488
Uvira	UV2_74	3504	110	0,03	46,32	2.628.348
Uvira	UV2_75	3504	1264	0,36	13,58	1.043.978
Uvira	UV2_76	3504	922	0,26	16,92	1.249.800
Uvira	UV2_77	3504	82	0,02	41,19	2.902.902
Uvira	UV2_78	3504	76	0,02	44,2	2.518.370
Uvira	UV2_79	3504	247	0,07	33,02	2.100.552
Uvira	UV2_80	3504	258	0,07	27,63	1.686.586
Uvira	UV2_81	3504	91	0,03	36,43	2.291.492
Uvira	UV2_82	3504	1415	0,4	11,92	1.042.068
Uvira	UV2_83	3504	382	0,11	29,49	1.852.228

Uvira	UV2_85	3504	421	0,12	25,94	1.485.770
Uvira	UV2_86	3504	613	0,17	26,61	1.975.046
Uvira2	UV3_106	3504	1175	0,34	14,33	1.165.406
Uvira2	UV3_113	3504	528	0,15	21,29	1.356.800
Uvira2	UV3_115	3504	1026	0,29	15,56	1.195.774
Uvira2	UV3_116	3504	279	0,08	30,25	2.202.068
Uvira2	UV3_117	3504	1257	0,36	18,21	1.187.018
Uvira2	UV3_118	3504	1674	0,48	10,35	1.086.542
Uvira2	UV3_39	3504	238	0,07	39,84	2.457.806
Uvira2	UV3_40	3504	313	0,09	29,18	1.653.016
Uvira2	UV3_41	3504	74	0,02	37,18	2.246.128
Uvira2	UV3_42	3504	148	0,04	31,49	1.937.298
Uvira2	UV3_43	3504	71	0,02	38,68	2.476.646
Uvira2	UV3_44	3504	110	0,03	38,29	2.160.290
Uvira2	UV3_45	3504	745	0,21	19,35	1.435.792
Uvira2	UV3_46	3504	194	0,06	29,02	1.723.056
Uvira2	UV3_48	3504	329	0,09	22,84	1.398.694
