# Supplementary figures and images for: First genomic study on Lake Tanganyika sprat Stolothrissa tanganicae: a lack of population structure calls for integrated management of this important fisheries target species
Source: BMC Evol Biol. 2019 Jan 8;19:6. doi: 10.1186/s12862-018-1325-8 (PMC6323704; doi:10.1186/s12862-018-1325-8)

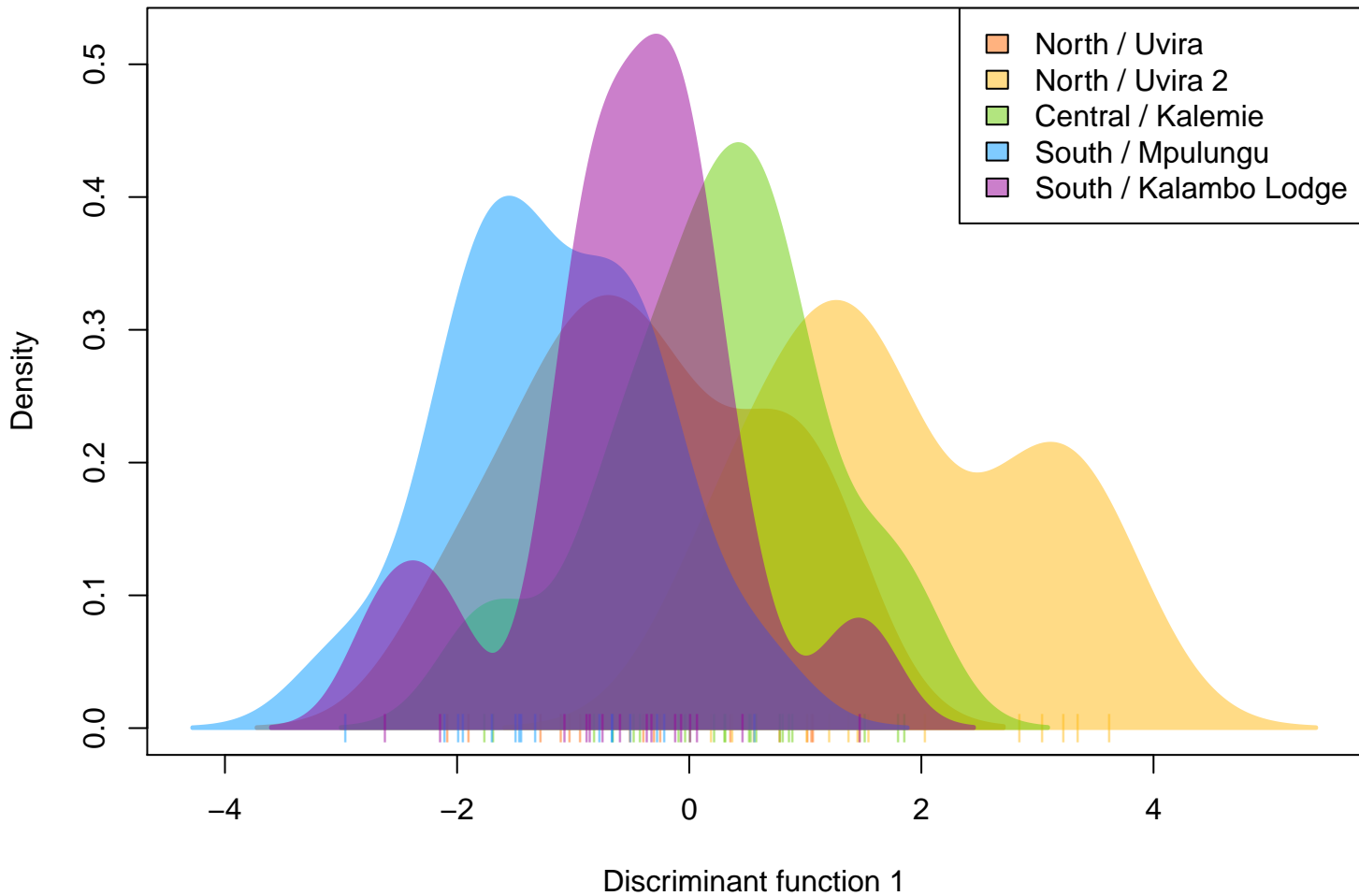

Supplement: Supplementary file 2 — Density plot of DAPC. Densities of individuals on the first discriminant function of the DAPC shown in Fig. 4. (PDF 28 kb) [file 12862_2018_1325_MOESM2_ESM.pdf]

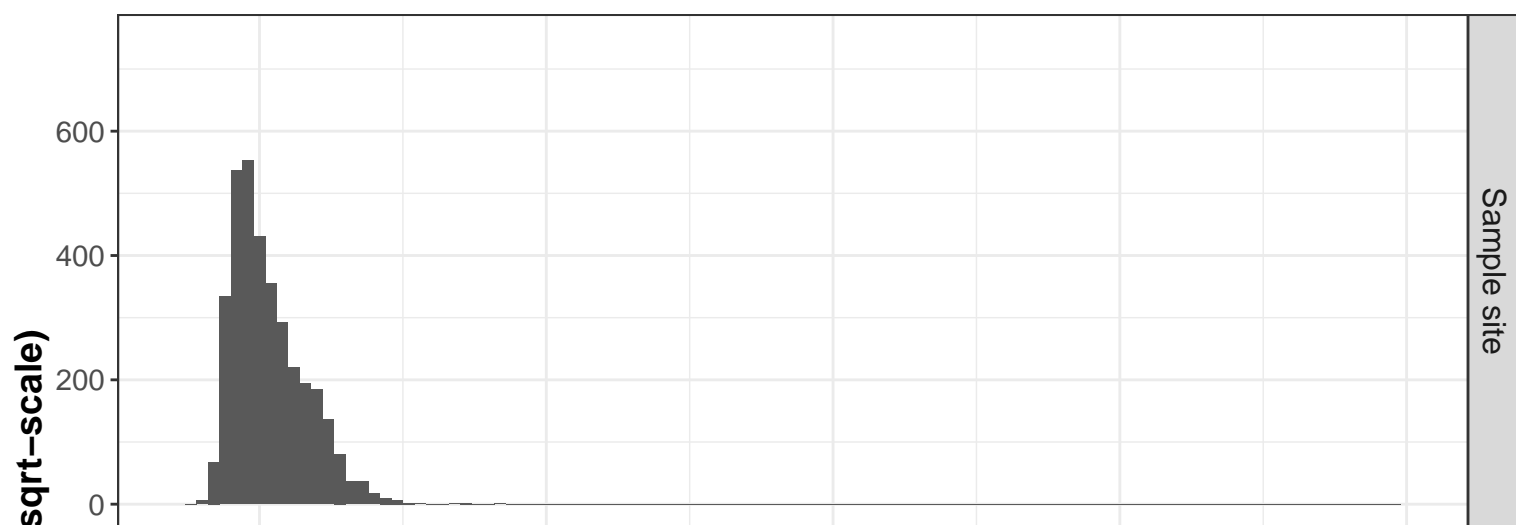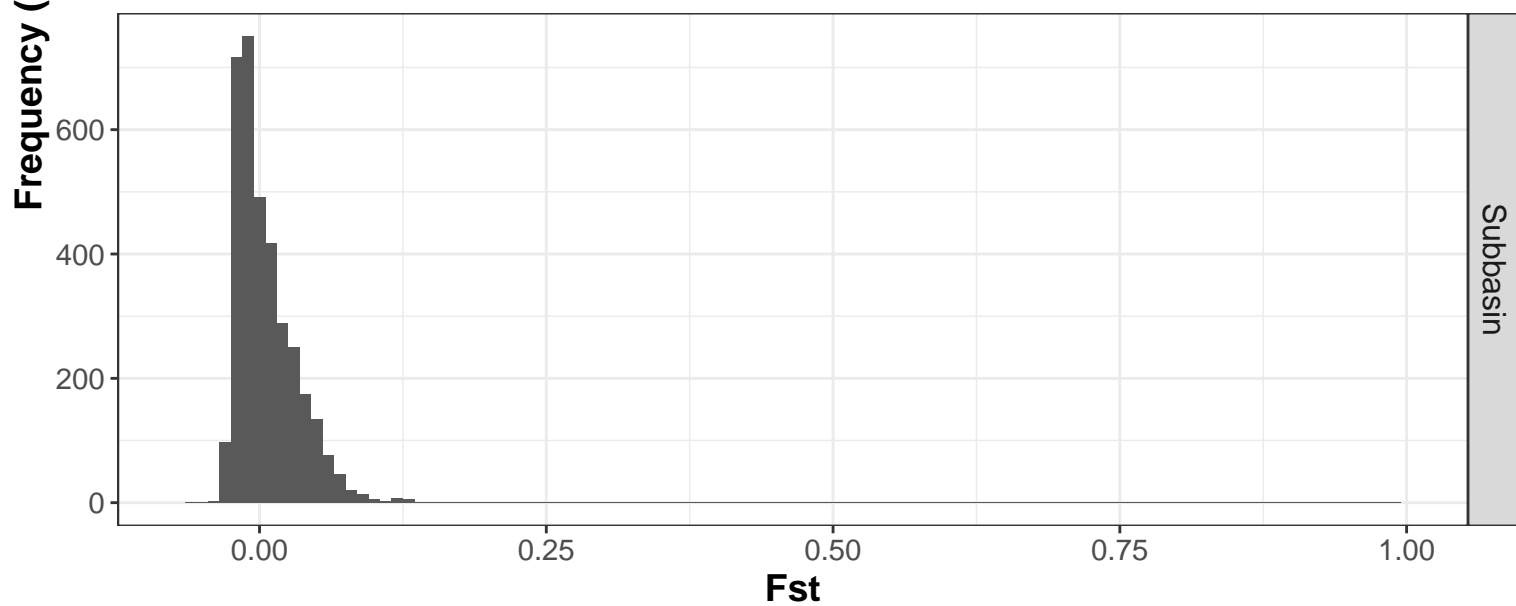

Supplement: Supplementary file 3 — Frequency distribution of global FST of Stolothrissa tanganicae per SNP. Grouping by sampling site and subbasin. (PDF 5 kb) [file 12862_2018_1325_MOESM3_ESM.pdf]

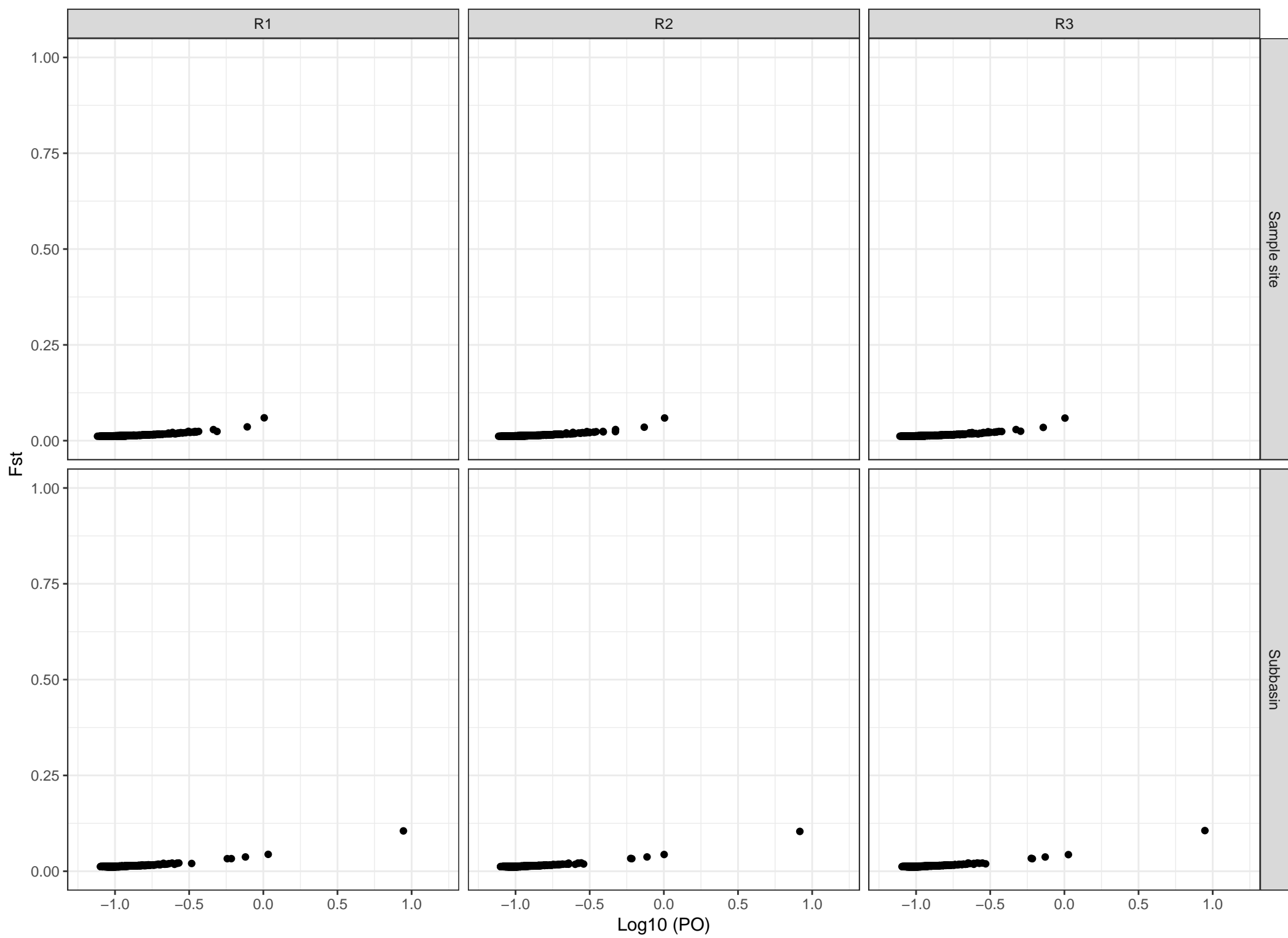

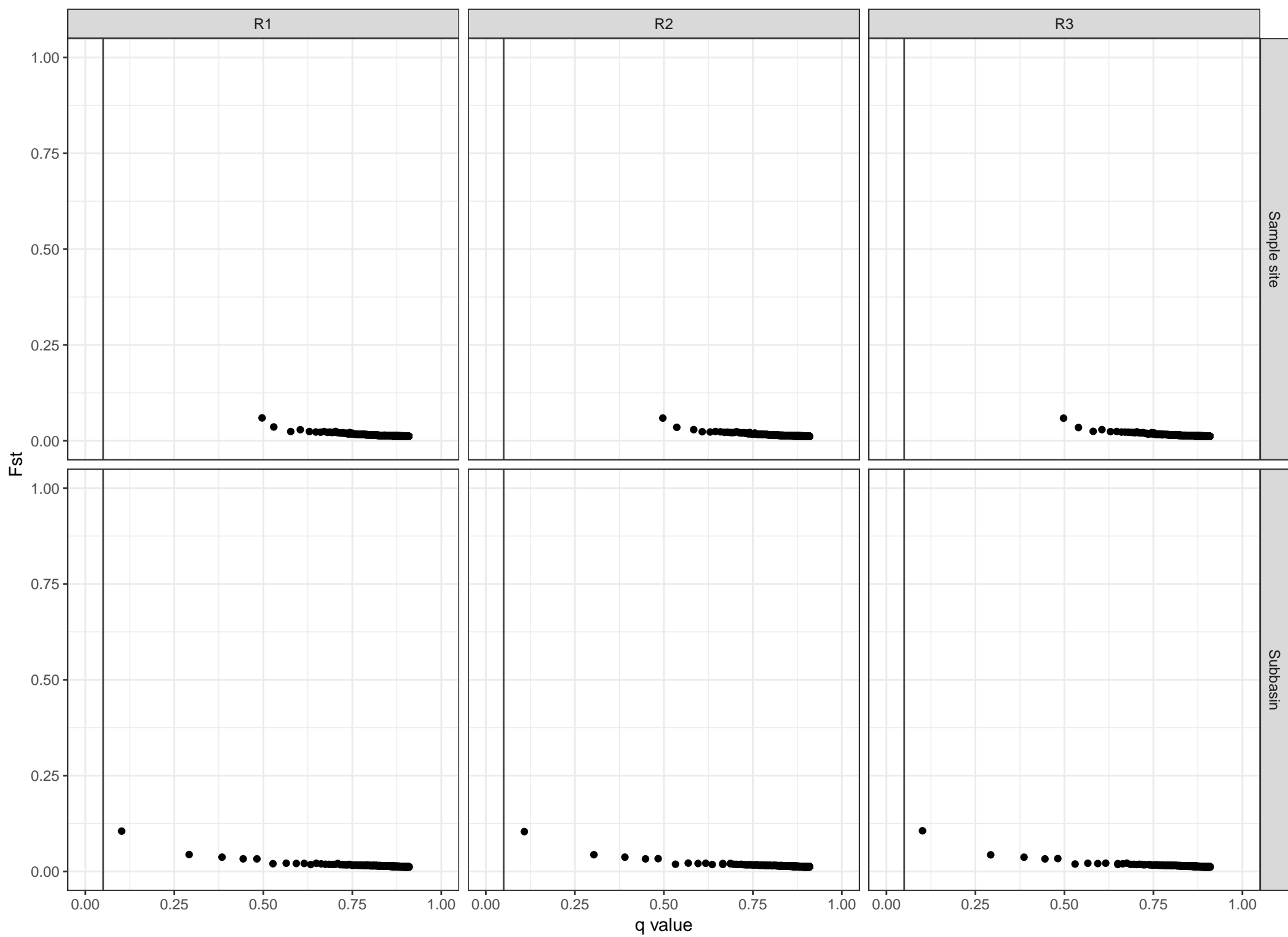

Supplement: Supplementary file 4 — Outlier analysis based on BayeScan v2.1. A. FST-Log10 posterior probability for two levels (sampling site and subbasin) for each of the three replicates (R1, R2, R3). B. Q value for grouping according sampling site and subbasin with each of the three replicates (R1, R2, R3). (PDF 169 kb) [file 12862_2018_1325_MOESM4_ESM.pdf]

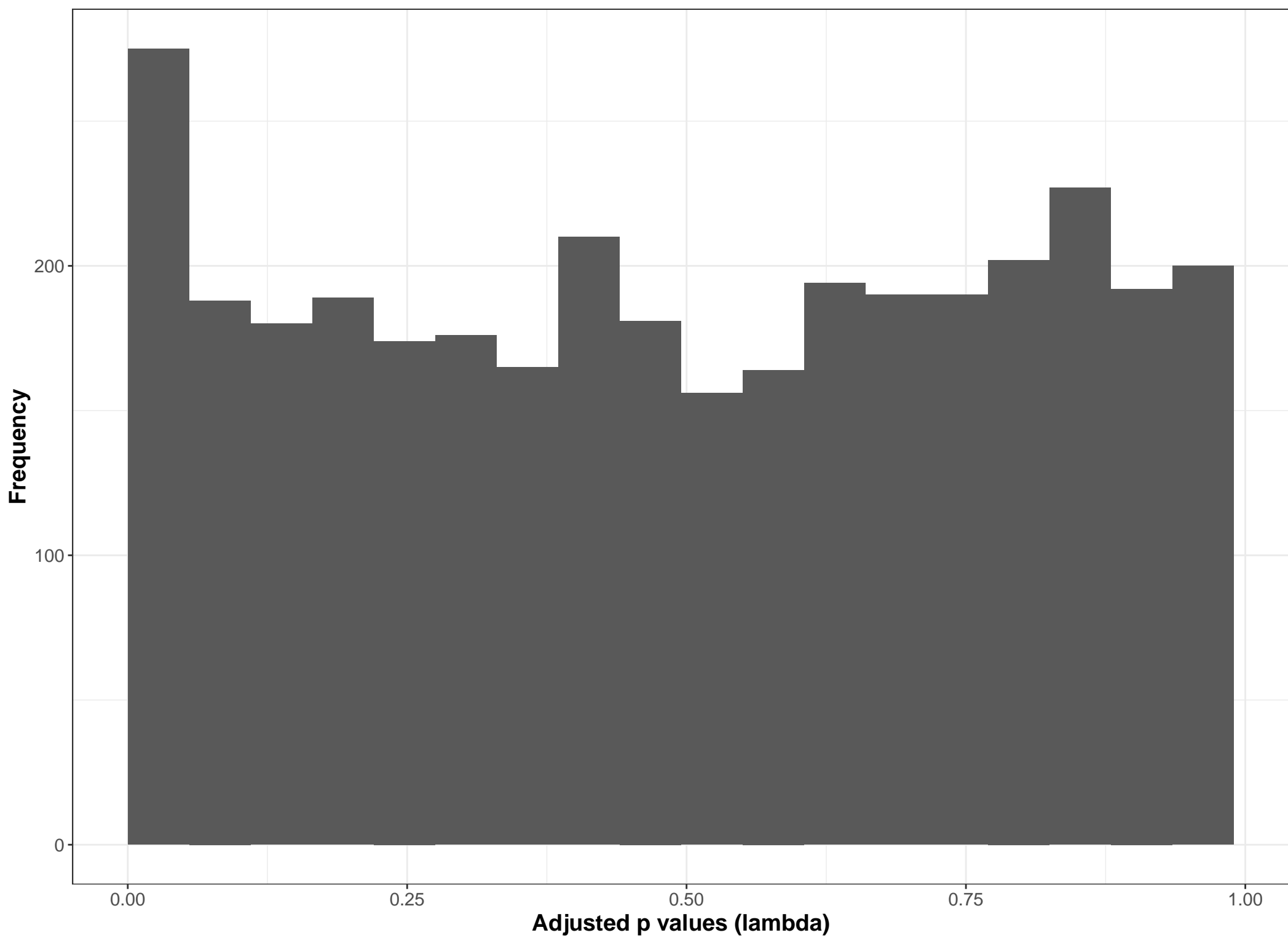

Supplement: Supplementary file 5 — Individual-based latent fixed mixed model (LFMM) analysis. Distribution of adjusted p-values, corrected with the genomic inflation factor. Made with the LEA package in R. (PDF 4 kb) [file 12862_2018_1325_MOESM5_ESM.pdf]
